# Supplementary material for: A Global Screen for Assembly State Changes of the Mitotic Proteome by SEC-SWATH-MS
Source: Cell Syst. 2020 Feb 26;10(2):133–155.e6. doi: 10.1016/j.cels.2020.01.001 (PMC7042714; doi:10.1016/j.cels.2020.01.001)

P04920 | B3A2_HUMAN | SLC4A2 AE2 EPB3L1 HKB3 MPB3L
Monomer MW [kDa]: 137.009 Monomer expected elution fraction: 37

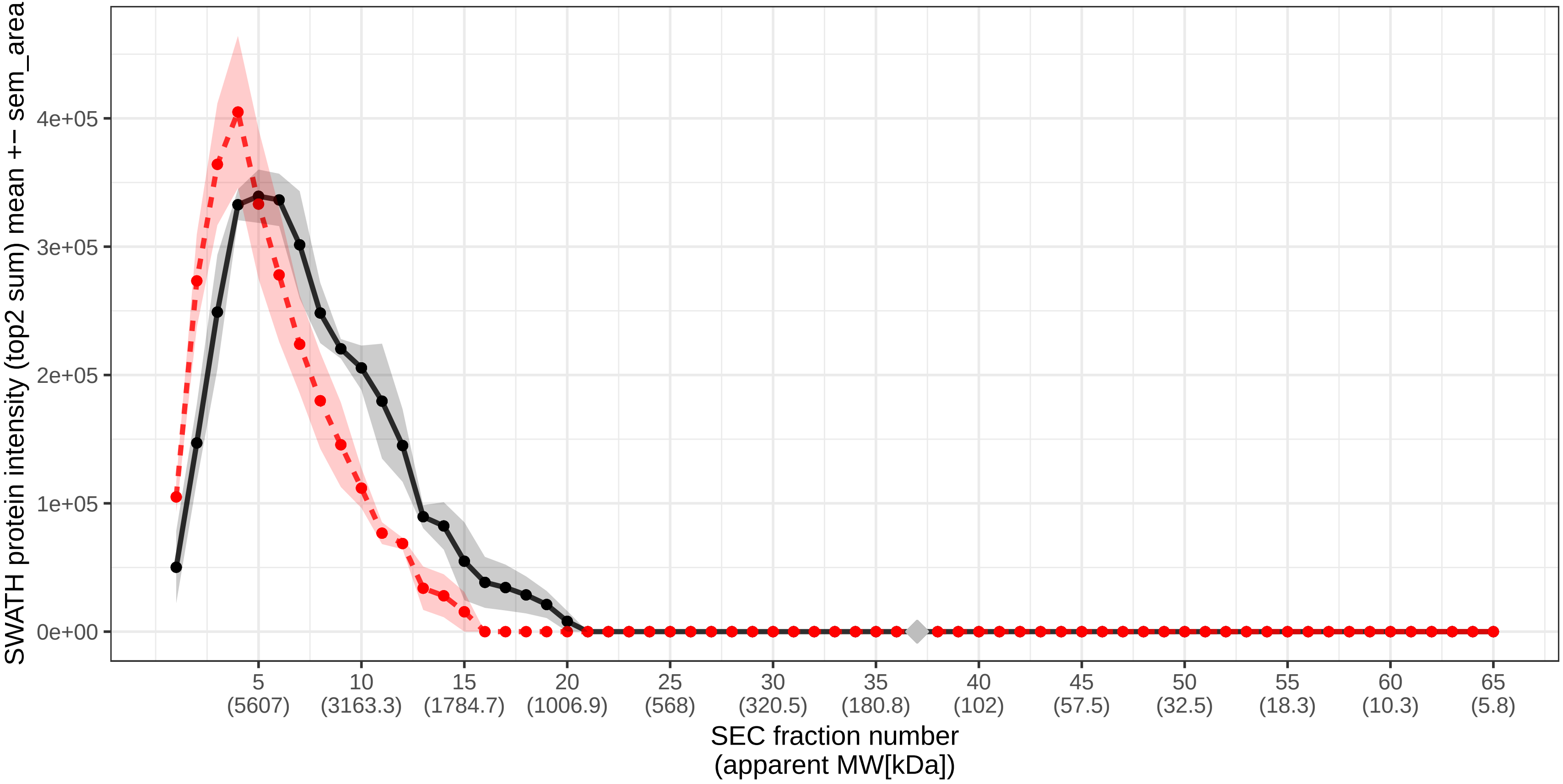

Supplement: Data S1. SEC-SWATH-MS Protein Chromatograms, Related to Figure 1 [file mmc6.zip › SECchrom_P04920_B3A2_HUMAN_SLC4A2_AE2_EPB3L1_HKB3_MPB3L.pdf]
